# Adaptation and Mal-Adaptation to Ambient Hypoxia; Andean, Ethiopian and Himalayan Patterns

**DOI:** 10.1371/journal.pone.0002342

**Published:** 2008-06-04

**Authors:** Guoqiang Xing, Clifford Qualls, Luis Huicho, Maria River-Ch, Tsering Stobdan, Marat Slessarev, Eitan Prisman, Soji Ito, Hong Wu, Angchuk Norboo, Diskit Dolma, Moses Kunzang, Tsering Norboo, Jorge L. Gamboa, Victoria E. Claydon, Joseph Fisher, Guta Zenebe, Amha Gebremedhin, Roger Hainsworth, Ajay Verma, Otto Appenzeller

**Affiliations:** 1 Department of Psychiatry, Uniformed Services University of the Health Sciences, Bethesda, Maryland, United States of America; 2 Department of Mathematics and Statistics, University of New Mexico, Albuquerque, New Mexico, United States of America; 3 Departamento Académico de Pediatría, Universidad Nacional Mayor de San Marcos, Lima, Peru; 4 Departamento de Ciencias Biológicas, Facultad de Ciencias y Filosofía, Instituto de Investigaciones de Altura, Universidad Peruana Cayetano Heredia, Lima, Peru; 5 Ladakh Institute of Prevention and Ladakh Heart Foundation, Leh, Ladakh, India; 6 Department of Anesthesiology, University of Toronto, Toronto, Canada; 7 Department of Physiology, University of Toronto, Toronto, Canada; 8 Department of Anesthesiology, Nagoya City University Graduate School of Medical Sciences, Nagoya, Japan; 9 Department of Medical Crisis Management, Nagoya City University Graduate School of Medical Sciences, Nagoya, Japan; 10 Department of Pharmacology, Uniformed Services University of the Health Sciences, Bethesda, Maryland, United States of America; 11 Sonam Norboo Memorial Hospital Leh, Ladakh, India; 12 Ladakh Autonomous Hill Development Council Leh, Ladakh, India; 13 Department of Physiology, University of Kentucky, Louisville, Kentucky, United States of America; 14 Department of Neurology, University of Kentucky, Louisville, Kentucky, United States of America; 15 School of Kinesiology, Simon Fraser University, Burnaby, British Columbia, Canada; 16 Department of Neurology, University of Addis Ababa and Yehuleshet Higher Clinic, Addis Ababa, Ethiopia; 17 Department of Medicine, University of Addis Ababa, Addis Ababa, Ethiopia; 18 Institute for Cardiovascular Research, University of Leeds, Leeds, United Kingdom; 19 Department of Neurology, Uniformed Services University of the Health Sciences, Bethesda, Maryland, United States of America; 20 Department of Neurology, New Mexico Health Enhancement and Marathon Clinics Research Foundation, Albuquerque, New Mexico, United States of America; University of Arkansas, United States of America

## Abstract

The study of the biology of evolution has been confined to laboratories and model organisms. However, controlled laboratory conditions are unlikely to model variations in environments that influence selection in wild populations. Thus, the study of “fitness” for survival and the genetics that influence this are best carried out in the field and in matching environments.

Therefore, we studied highland populations in their native environments, to learn how they cope with ambient hypoxia. The Andeans, African highlanders and Himalayans have adapted differently to their hostile environment.

Chronic mountain sickness (CMS), a loss of adaptation to altitude, is common in the Andes, occasionally found in the Himalayas; and absent from the East African altitude plateau.

We compared molecular signatures (distinct patterns of gene expression) of hypoxia-related genes, in white blood cells (WBC) from Andeans with (n = 10), without CMS (n = 10) and sea-level controls from Lima (n = 20) with those obtained from CMS (n = 8) and controls (n = 5) Ladakhi subjects from the Tibetan altitude plateau. We further analyzed the expression of a subset of these genes in Ethiopian highlanders (n = 8). In all subjects, we performed the studies at their native altitude and after they were rendered normoxic.

We identified a gene that predicted CMS in Andeans and Himalayans (PDP2). After achieving normoxia, WBC gene expression still distinguished Andean and Himalayan CMS subjects.

Remarkably, analysis of the small subset of genes (n = 8) studied in all 3 highland populations showed normoxia induced gene expression changes in Andeans, but not in Ethiopians nor Himalayan controls. This is consistent with physiologic studies in which Ethiopians and Himalayans show a lack of responsiveness to hypoxia of the cerebral circulation and of the hypoxic ventilatory drive, and with the absence of CMS on the East African altitude plateau.

## Introduction

The human genome was sequenced only recently, but already the focus of much research has shifted to human genetic variation and epigenomics has been selected as one of NIH's Roadmaps Initiative for 2008 [1] The discovery of numerous diseases associated with DNA duplications and deletions, with single nucleotide polymorphisms (SNPs), and with gene mutations has set the stage for the analysis of the molecular underpinnings of adaptation to life at high altitudes [2]. The genomic era has also opened the way to studying the basis of susceptibility to chronic mountain sickness (CMS), a disease of maladaptation to altitude [3]. CMS has numerous parallels with diseases frequently encountered at sea level in which hypoxia plays an important role, such as chronic obstructive pulmonary disease, sleep apnea and cancer. Thus, lessons from the mountains might impact the understanding and treatment of diseases at sea level.

More than 140 million people live and work at altitudes above 2500 m [4]. The three large high altitude populations are the Andeans, the Himalayans and the lesser studied inhabitants of the of East African high altitude plateau. These populations have different phenotypes and different strategies for coping with their inhospitable hypoxic environment [5], [6]. However, their genotypes and the molecular signatures (defined as a distinct pattern of gene expression) that contribute to adaptation or maladaptation of these people remain poorly understood [7].

All highlanders are exposed to high levels of free radicals and oxidants [8]. These chemical species, byproducts of mitochondrial metabolism, include the superoxides (O_2_^−^), peroxides (H_2_O_2_) and hydroxyl radicals (OH^−^), and are collectively designated as reactive oxygen species (ROS ). ROS have also been associated with neurodegenerations such as Alzheimer's disease, heart disease and cancer, and they have been implicated in aging [9].

These molecules are also crucial for the maintenance of homeostasis. The ROS are abundantly formed during hypoxia and can serve as signaling molecules that help maintain homeostasis. Specific proteins such as the guanosine 3′,5′-monophosphate (cGMP)-dependent protein kinase PKG 1α isoform, may in fact serve as direct ROS sensors [10].

Here we examine a set of hypoxia-related genes expressed in white blood cells (WBC) in Andean highlanders born and living at 4338 m, both healthy subjects and patients suffering from CMS. We also collected WBC from the same individuals after a short sea level sojourn (in Lima, Peru at 150 m) and compared the gene expression profiles with those from permanent sea level inhabitants in Lima.

We studied Himalayan highlanders living at 4450 m. on the Tibetan plateau; healthy natives from a village named Korzok and their neighbors with CMS. Because of the difficulties in rapidly getting people off the Tibetan plateau to low altitude we exposed both group to hyperoxia for one hour at their native altitude. We obtained WBC from this cohort before and after hyperoxia.

We conducted a parallel study, on a subset of the genes in Ethiopian highlanders. We obtained two samples of WBC, one at their resident altitude of 3622 m in the Simen Mountain National Park and one within 24 hours of arrival in the Tekeze River Gorge (794 m.). For genes studied, see Text S1.

## Results

We examined differences in gene expression in high altitude Peruvians with CMS and in healthy Peruvian high altitude subjects who were free of symptoms and signs of CMS (CMS-sc of <12, our normal high altitude controls in Peru). Expression levels in these two Peruvian altitude-dwelling populations, at altitude and after they were brought down to Lima, were then compared to those in sea-level Peruvians at Lima. We next compared our Peruvian results with those we obtained from Himalayan subjects with and without CMS, in ambient air and after they were rendered hyperoxic, with those from Ethiopians, a high altitude population in whom CMS has not been reported. The demographics of our Peruvian, Himalayan and Ethiopian study groups are given in Table 1.

**Table 1:** Demographics of our study populations. (mean±SD).

	ETH. HIGH	PERU CONTR. HIGH	ETH. LOW	PERU CONTR. LOW	HIMALAYAN CONTR. AMBIENT	HIMALAYAN CONTR. HYPEROXIA.
**AGE**	**35.5±4§**	**37.1±6.7**	…..	…..	**42.1±9.4**	……
**O_2_ SAT**	**88±3.2§**	91.4±1.9*	**96.7±1.4**	**99.9±0.3**	**81.8±4.4**	**98.2±0.7**
**P_et_CO_2_(mm.Hg)**	**37.1±2.3§**	31.8±3.5§**	**47.1±4.4§**	**39.3±2.2§**	**29.9±3.7**	NA
**CMS-score**	**0.3±0.5§**	**1.3±1.6§**	NA	NA	**8.2±3.4**	NA
**Hematocrit %**	**48.5±4.6§**	**54.6±2.4§** †	NA	**53.6±4.6**	**54.1±5.7** †	NA

Values are reported either at high altitude (HIGH), or low altitude (LOW) in Peru and Ethiopia. The Himalayans were studied at the same altitude in ambient air (ambient) and after 1 hour of hyperoxia. ^§^ From reference [22].

† P = 0.004. ^*^P = 0.02. ^**^P<0.01 compared to Ethiopians. …….. = no change; NA = not done.

To assess the impact of specific genes on the CMS scores, using a sliding scale of CMS-scores derived from both controls and CMS patients, we constructed an “impact table” for the Cerro de Pasco groups at altitude (Table 2) and in Lima Table 3.

**Table 2:** Impact table to show the predicting power of specific hypoxia-related genes on CMS (CMS-score≥12) in our Andean subjects in Cerro de Pasco, Peru, altitude 4338 m.

HIGH CERRO A			ADJ. for PDP2		Adj. PDP2	
*GENES*	STB	P	STB	P	STB	P
lg_EPO	0.05	0.82	0.09	0.66	−0.56	**0.012**
lg_HPH1	−0.02	0.94	−0.04	0.85	−0.56	**0.013**
lg_HPH2	−0.04	0.85	−0.01	0.95	−0.56	**0.013**
lg_HPH3	0.24	0.30	0.44	0.03	−0.69	**0.002**
lg_VEGFC	−0.05	0.83	0.33	0.16	−0.73	**0.005**
lg_PDK1	−0.19	0.42	0.00	0.11	−0.56	**0.019**
lg_PDP1	−0.43	0.05	−0.03	0.93	−0.54	**0.101**
lg_HIF1A	0.01	0.95	0.21	0.32	−0.62	**0.008**
lg_HIF1B	−0.18	0.45	0.28	0.28	−0.73	**0.009**
lg_HIF2A	−0.37	0.11	−0.10	0.7	−0.51	**0.048**
lg_HIF3A	−0.28	0.24	0.16	0.56	−0.66	**0.023**
lg_PDK2	−0.45	0.05	−0.16	0.54	−0.46	**0.095**
**lg_PDP2**	**−0.56**	**0.01**	…	…	…	**…**
lg_PDK3	−0.50	0.02	−0.27	0.3	−0.40	**0.12**
lg_PDK4	−0.39	0.09	−0.13	0.6	−0.49	**0.053**
lg_PDHE1A1	−0.28	0.23	0.20	0.7	−0.70	**0.021**
lg_GAPDH	0.17	0.46	0.17	0.41	−0.55	**0.012**
lg_EPOR	0.12	0.62	0.29	0.15	−0.64	**0.005**
lg_GLUT1	0.04	0.86	0.10	0.64	−0.57	**0.012**
lg_LDHA	MISSING					
lg_CATD	MISSING					

To assess the impact of specific genes on chronic mountain sickness-score (CMS-sc), using a sliding scale (continuous), we constructed an “impact table” for the combined (CMS & Controls) Cerro de Pasco groups at altitude (A).

In univariate linear regression model, **PDP2** is found to be the most significant predictor of CMS-sc (highlighted). Adjusting for **PDP2** eliminates the impact of the other genes on the CMS-sc (columns, ADJ for PDP2). However, adjusting PDP2 for each of the remaining genes, assayed here, strengthens the impact of PDP2 (columns, Adj. PDP2).

This supports the importance of **PDP2** in predicting CMS-sc.

**PDP2** is the phosphatase that de-phosphorylates the E1-alpha subunit of pyruvate dehydrogenase which promotes pyruvate entry into the Krebs cycle,

The **STB** is the standardized estimate for the parameter estimate of an explanatory variable in the logistic regression model and is computed by

multiplying the estimate by the sample standard deviation for the explanatory variable and dividing by π/√3 [Ref. SAS 9.1; On Line Help] (For glossary of terms see Text S1).

**Table 3:** Impact table to show the predicting power of specific hypoxia-related genes on CMS (CMS-score≥12) in our Andean subjects in Lima, Peru, altitude 150 m.

LOW LIMA B			ADJ. for PDP2		Adj. PDP2	
*GENES*	STB	P	STB	P	STB	P
lg_EPO	−0.28	0.23	0.02	0.91	−0.73	**<0.0001**
lg_HPH1	−0.28	0.27	−0.17	0.36	−0.68	**0.002**
lg_HPH2	−0.12	0.63	0.21	0.25	−0.81	**<0.0004**
lg_HPH3	−0.16	0.64	0.18	0.44	−0.87	**0.004**
lg_VEGFC	MISSING					
lg_PDK1	−0.60	0.005	−0.32	0.08	−0.56	**0.005**
lg_PDP1	−0.37	0.11	−0.10	0.6	−0.69	**0.001**
lg_HIF1A	−0.21	0.34	−0.02	0.9	−0.72	**<0.0004**
lg_HIF1B	−0.31	0.18	0.12	0.6	−0.79	**<0.0004**
lg_HIF2A	−0.50	0.02	−0.05	0.81	−0.69	**0.006**
lg_HIF3A	−0.28	0.27	−0.17	0.36	−0.68	**0.002**
lg_PDK2	−0.37	0.11	−0.17	0.35	−0.68	**0.001**
**lg_PDP2**	**−0.73**	**0.0003**	…	…	…	**…**
lg_PDK3	−0.45	0.05	−0.12	0.55	−0.67	**0.003**
lg_PDK4	0.21	0.38	0.12	0.46	−0.71	**<0.0004**
lg_PDHE1A1	−0.67	0.001	−0.35	0.1	−0.51	**0.021**
lg_GAPDH	−0.16	0.65	0.18	0.44	−0.87	**0.004**
lg_EPOR	−0.36	0.12	0.01	0.95	−0.73	**0.002**
lg_GLUT1	MISSING					
lg_LDHA	−0.69	0.0008	−0.37	0.08	−0.48	**0.03**
lg_CATD	−0.49	0.03	−0.15	0.44	−0.65	**0.004**

To assess the impact of specific genes on chronic mountain sickness-score (CMS-sc), using a sliding scale (continuous), we constructed an “impact table” for the combined (CMS & Controls) Cerro de Pasco groups while normoxic in Lima (sea level) (**B**).

In univariate linear regression model, **PDP2** is found to be the most significant predictor of CMS-sc (highlighted) even in normoxia. Adjusting for **PDP2** eliminates the impact of the other genes on the CMS-sc (columns, ADJ for PDP2). However, adjusting PDP2 for each of the remaining genes, assayed here, strengthens the impact of PDP2 (columns, Adj. PDP2). This supports the importance of **PDP2** in predicting the CMS-scores of our subjects while in Cerro de Pasco at altitude. (For glossary of terms see table 2 and Text S1).

A linear regression model shows that the best predictor of CMS (highlighted in the tables) in this population is the expression of PDP2, a gene encoding a protein phosphatase that de-phosphorylates the E1-alpha subunit of pyruvate dehydrogenase and thus promotes pyruvate entry into the Krebs cycle. Adjusting for PDP2 eliminates the impact of the other genes on the CMS-sc (columns, ADJ for PDP2). However, adjusting for each of the remaining genes strengthens the impact of PDP2 (columns, Adj. PDP2). This supports the importance of PDP2 in predicting CMS.

We constructed a similar impact table for our Himalayan subjects (Table 4) while at their resident altitude breathing ambient air. The best predictor of CMS in a linear regression model in the Himalayas is also PDP2. However, after 1 hour of hyperoxia, at the same altitude, the best predictor of CMS is PDK4 (Table 5).

**Table 4:** Impact table to show the predicting power of specific hypoxia-related genes on CMS (CMS-score≥12) in the Himalayas. (Korzok village altitude 4450 m.).

In Himalayas				Adjust for PDP2			Adjust PDP2	
A	STB	P		STB	P		STB	P
PDK1	−0.42	0.18	PDK1	0.19	0.74	PDP2	−0.70	0.23
PDK2	0.14	0.67	PDK2	0.51	0.09	PDP2	−0.79	0.02
PDK3	−0.39	0.21	PDK3	0.43	0.48	PDP2	−0.92	0.15
PDK4	0.24	0.46	PDK4	0.37	0.19	PDP2	−0.62	0.04
PDP1	−0.34	0.28	PDP1	−0.07	0.84	PDP2	−0.51	0.16
**PDP2**	−0.54	0.07		**…**	**…**		**…**	**…**
PDHE1a1	−0.26	0.41	PDHE1a1	0.42	0.35	PDP2	−0.87	0.07
HIF1a	0.29	0.36	HIF1a	0.57	0.04	PDP2	−0.75	0.01
HIF1b	0.16	0.62	HIF1b	0.87	0.00	PDP2	−1.10	0.00
HIF2a	−0.02	0.96	HIF2a	0.64	0.07	PDP2	−0.97	0.01
HIF3a	−0.04	0.89	HIF3a	0.54	0.13	PDP2	−0.89	0.02
HPH1	0.09	0.78	HPH1	0.41	0.18	PDP2	−0.73	0.03
HPH2	−0.25	0.43	HPH2	0.09	0.79	PDP2	−0.60	0.12
HPH3	0.37	0.24	HPH3	0.18	0.55	PDP2	−0.47	0.15
VEGF_R2	−0.26	0.42	VEGF_R2	0.22	0.58	PDP2	−0.69	0.10
EPOS	0.50	0.10	EPOS	0.55	0.03	PDP2	−0.59	0.02
CAT_D	0.20	0.54	CAT_D	0.40	0.16	PDP2	−0.66	0.03
GLUT1	0.20	0.54	GLUT1	0.38	0.18	PDP2	−0.65	0.03
GAPDH	0.32	0.32	GAPDH	0.35	0.20	PDP1	−0.57	0.05
GAPDH	0.32	0.32	GAPDH	0.35	0.20	PDP2	−0.57	0.05

To assess the impact of specific genes on chronic mountain sickness-score (CMS-sc), using a sliding scale (continuous), we show an “impact table” for the combined (CMS & Controls) Himalayan (Ladakh) groups at native altitude **(A).**

In univariate linear regression model, **PDP2** is found to be the most significant predictor of CMS-sc (highlighted). Adjusting for PDP2 eliminates the impact of the other genes on the CMS-sc (columns, ADJ for PDP2). However, adjusting PDP2 for each of the remaining genes, assayed here, strengthens the impact of PDP2 (columns, Adj. PDP2). This supports the importance of **PDP2** in predicting CMS-sc in the Himalayas just as in the Andes (For glossary of terms, see table 2 and Text S1).

**Table 5:** Impact table to show the predicting power of specific hypoxia-related genes on CMS (CMS-score≥12) in the Himalayas (Korzok village altitude 4450 m.) after 1 hour of hyperoxia.

				Adjust for PDK4			Adjust PDK4	
B	STB	P		STB	P		STB	P
**PDK1**	−0.35	0.25	PDK1	−0.18	0.50	PDK4	0.54	**0.06**
**PDK2**	0.06	0.86	PDK2	0.27	0.31	PDK4	0.68	**0.02**
**PDK3**	−0.22	0.47	PDK3	−0.17	0.51	PDK4	0.58	**0.04**
**PDK4**	**0.60**	**0.03**	**PDK4**	…	…	…	…	**…**
**PDP1**	−0.42	0.15	PDP1	−0.29	0.27	PDK4	0.52	**0.06**
**PDP2**	−0.15	0.62	PDP2	−0.12	0.66	PDK4	0.59	**0.04**
**PDHE1a1**	−0.45	0.13	PDHE1a1	−0.36	0.15	PDK4	0.54	**0.04**
**HIF1a**	−0.39	0.19	HIF1a	−0.49	0.04	PDK4	0.67	**0.01**
**HIF1b**	−0.20	0.51	HIF1b	−0.22	0.39	PDK4	0.60	**0.03**
**HIF2a**	−0.58	0.04	HIF2a	−0.46	0.07	PDK4	0.47	**0.06**
**HIF3a**	−0.51	0.08	HIF3a	−0.42	0.09	PDK4	0.52	**0.04**
**HPH1**	−0.32	0.29	HPH1	−0.31	0.22	PDK4	0.59	**0.03**
**HPH2**	−0.28	0.35	HPH2	−0.20	0.43	PDK4	0.57	**0.05**
**HPH3**	0.10	0.75	HPH3	−0.19	0.51	PDK4	0.67	**0.03**
**VEGF_R2**	0.11	0.72	VEGF_R2	−0.06	0.81	PDK4	0.61	**0.04**
**EPOS**	−0.03	0.92	EPOS	−0.04	0.86	PDK4	0.60	**0.04**
**CAT_D**	0.02	0.95	CAT_D	−0.09	0.74	PDK4	0.61	**0.04**
**GLUT1**	−0.25	0.42	GLUT1	−0.22	0.38	PDK4	0.59	**0.04**
**VEGF_C**	−0.08	0.79	VEGF_C	−0.14	0.60	PDK4	0.61	**0.04**
**GAPDH**	−0.14	0.65	GAPDH	−0.10	0.69	PDK4	0.59	**0.04**

To assess the impact of specific genes on chronic mountain sickness-score (CMS-sc), using a sliding scale (continuous), we show an “impact table” for the combined (CMS & Controls) Himalayan (Ladakh) groups at native altitude after 1 hour of hyperoxia **(B)**. The best predictor, under these circumstances, is **PDK4**.

In univariate linear regression model, **PDK4** is found to be the most significant predictor of CMS-sc (highlighted). Adjusting for PDK4 eliminates the impact of the other genes on the CMS-sc (columns, ADJ for PDK4). However, adjusting PDK4 for each of the remaining genes, assayed here, strengthens the impact of PDK4 (columns, Adj.PDK4). This supports the importance of PDK4 in predicting CMS-sc in the Himalayas under conditions of hyperoxia just as in the Andes in normoxia, see Table 3, (For glossary of terms, see table 2 and Text S1).

**PDK4** (pyruvate dehydrogenase Kinase 4) is a kinase involved in “aerobic glycolysis” (the Warburg effect). It supports a metabolic pattern seen in many (but not all) hypoxia adapted tissues, also in cancer cells and activated immune cells.

**Figure 1:**
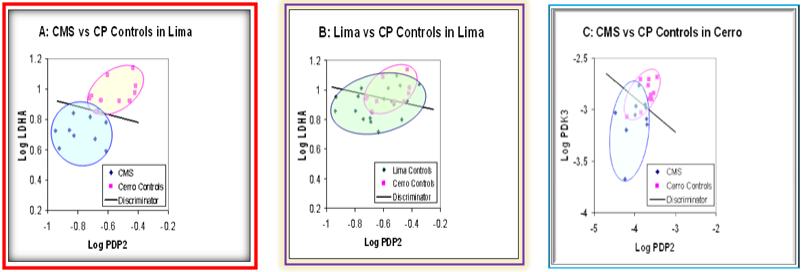
Niche (scatter) plots of genes that best predict the presence of CMS in Peruvian altitude dwellers. Because LDHA was not assayed at altitude it was substituted by PDK3 which was highly correlated with LDHA. A: CMS vs CP Controls in Lima (normoxia). B: Lima Controls vs CP Controls in Lima. C: CMS vs CP Controls in Cerro de Pasco (4338 m,). When hypoxia was shown to have been replaced by short sea level sojourn, normoxia (A), the CMS patients were clearly separated from control altitude dwellers. B. There were no differences between altitude controls and Lima controls in Lima. In Cerro de Pasco the CMS patients and controls were mostly separated by the “discriminator line”. Discriminator line based on discrimination of the two groups by logistic regression. CMS = chronic mountain sickness. LDHA = Lactate dehydrogenase A. PDK3 = . Pyruvate dehydrogenase Kinase3. This kinase is involved in “aerobic glycolysis” (the Warburg effect). It suports a metabolic pattern seen in many (but not all) hypoxia adapted tissue, also in cancer cells and activated immune cells. PDP2 = phosphatase that de-phosphorylates the E1-alpha subunit of pyruvate dehydrogenase. CP = Cerro de Pasco altitude 4338 m. Cerro = Cerro de Pasco. Con = Andean controls. H = high altitude. L = Low altitude

We further refined our analyses by constructing scatter plots, “niche” graphs, using genes that best explained the presence of CMS in a logistic regression analysis. We show the difference in gene expression between the 3 study groups in Lima and the 2 in Cerro de Pasco. In ambient normoxia of Lima, there was complete separation of the patients with CMS from the altitude controls as seen in the niche graphs. The controls, high-altitude and sea-level Lima natives, were indistinguishable in Lima. In their native altitude environment, however, the controls and CMS patients showed slight overlap of the niche graphs (Fig. 1).

**Figure 2:**
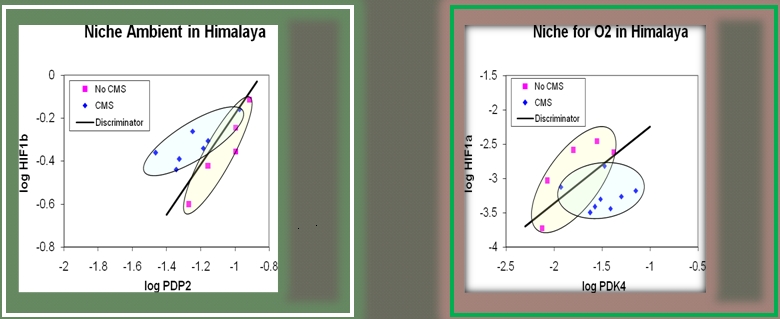
Niche (scatter) plots of genes that best predict the presence of CMS in Himalayan altitude dwellers. A. CMS vs Controls in Himalayans (Ladakh) at native altitude and ambient conditions at 4450 m. and at the same altitude after 1 hour of hyperoxia (B). A. In Korzok village, Ladakh, the CMS patients and controls were clearly separated by the “discriminator line”. B. When ambient hypoxia was shown to have been replaced by induced normoxia, the CMS patients were still separated from control altitude dwellers, although the best predictors for CMS had changed. Discriminator line based on discrimination of the two groups by logistic regression. CMS = chronic mountain sickness. PDK4 = . Pyruvate dehydrogenase Kinase 4. This kinase is involved in “aerobic glycolysis” (the Warburg effect).It supports a metabolic pattern seen in many (but not all) hypoxia adapted tissue, also in cancer cells and activated immune cells. PDP2 = phosphatase that de-phosphorylates the E1-alpha subunit of pyruvate dehydrogenase. HIF1a, HIF2a, HIF3a, HIF1b = Hypoxia Inducible Factors, composed of alpha and beta subunits. Only the alpha subunit protein levels are regulated by oxygen. HIF1b is used as a dimerizing partner by all three HIF1a's.

A similar analysis of our Himalayan cohort (Fig. 2) showed good separation in ambient hypoxia but the CMS patients were less well separated from controls in induced normoxia.

**Figure 3:**
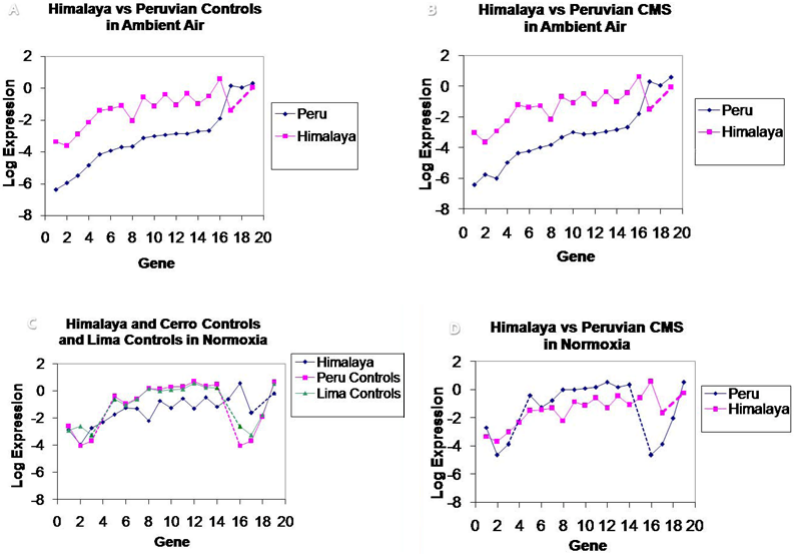
Quantitative comparisons of gene expression in Andean and Himalayan altitude dwellers. A. Controls in Peru altitude 4338 m. and Himalyan controls, on the Tibetan plateau in Ladakh, India, altitude 4450 m. B. CMS patients at the same altitudes as in A in Peru and in the Himalayas. C. Peruvian and Himalayan controls in normoxia. D. Peruvian and Himalayan CMS patients in normoxia. The genes compared in the 2 populations are: 1 = HIF-1a; 2 = HPH3; 3 = HIF-3a; 4 = VEGFC; 5 = PDK4; 6 = HIF2a; 7 = PDP2; 8 = PDK1; 9 = PDHE1a1; 10 = EPOR; 11 = PDP1; 12 = PDK3; 13 = HIF1b; 14 = PDK2; 15 = GLUT1; 16 = GAPDH; 17 = HPH1; 18 = EPO; 19 = HPH2. Interrupted lines span across genes not assayed. In ambient hypoxia all Himalayans (controls and CMS) had significantly higher gene expression when compared to Andeans except for HPH1. The differences between the groups, while normoxic, were not significant. (C. D.).

Quantitative comparisons of gene expressions of Andean and Himalayan subjects are shown in Fig. 3. All Himalayans (CMS patients and controls) had significantly higher expression levels of the genes analyzed (except for HPH1) than Andeans living at similar altitude.

**Figure 4:**
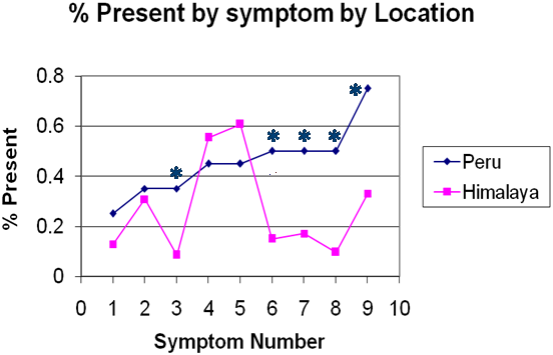
Symptoms (Signs) Number for CMS scores in the Peruvian Andes (4338 m. Peru) and Tibetan plateau (4450 m. Himalaya): 1-SaO2_ 2SD below normal for altitude; 2-Breathlesness, palpitations; 3-Sleep disturbances; 4-Cyanosis of lips, face or fingers; 5-dilated veins; 6-Paresthesias in fingers and/or toes; 7-Tinnitus; 8-Hb_2SD above normal for altitude; 9-Headache. *Denotes significant differences. In the Peruvian Andes the % of subjects having: sleep disturbances, paresthesias, tinnitus, high hemoglobin and headache were significantly higher*. Both populations were scored using an internationally accepted scoring system (≥12 = CMS)[11].

A comparison of the symptomatology between CMS patients in the Andes and Himalayas is shown in Fig. 4. Both populations were scored using an internationally accepted scoring system devised for the high Andes [11].

**Figure 5:**
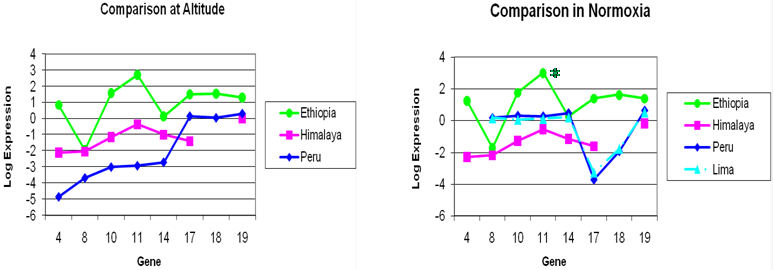
Expression signatures of a subset of genes examined in Ethiopians, Himalayans and Andeans. Comparisons at altitude (left) and in normoxia (right), same (control) subjects in each ethnic group. Note the lack of change in expression signatures with normoxia in the Ethiopians except for (PDP1, P<0.04). In Himalayans none of the gene expressions were changed by normoxia. No differences in molecular signatures between the Andean study groups (CMS and controls) except significant differences by location (normoxia in Lima). There were significant decreases in expression at altitude for most genes, except for HPH1, EPO and HPH2 in Andeans; VEGFC was not assayed in Andeans at low altitude. The genes were: #4-VEGFC; #8-PDK1; #10-EPOR; #11-PDP1; #14-PDK2; #17-HPH1; #18-EPO; #19-HPH2.

We examined a small subset of genes (n = 8) in our eight Ethiopian subjects. Most gene expressions were several orders of magnitude higher in Ethiopians than in Andeans and Himalayans at altitude. Remarkably, normoxia in the Tekeze River Gorge (794 m.) in Ethiopians, or normoxia in Himalayan controls had no effect on expression levels except for PDP1 which was significantly upregulated in normoxia (P<0.04). By contrast, in Peruvians, in Lima (normoxia), significant increases were found, except for HPH1, HPH2 and EPO which were down regulated (Fig. 5). Importantly, features of hypoxia-adapted tissues, such as up-regulation of enzymes related to “aerobic glycolysis” favoring lactate accumulation in the presence of hypoxia and reducing ROS formation (PDK1, PDK2), were significantly higher in Ethiopians in hypoxia than in Peruvians in their native hypoxic environment, suggesting that Andean tissues were poorly adapted to ambient hypoxia.

Genes involved in erythropoiesis were highly expressed in Ethiopians at altitude, and remained significantly higher in Ethiopians in normoxia (EPOR, EPO).

Vascular endothelial growth factor C (VEGFC), involved in blood vessel proliferation in response to hypoxia, was very high in Ethiopians at altitude and in the normoxic environment of the Gorge, but was significantly lower in Andeans and Himalayans at altitude. Unfortunately, it was not assayed in Peruvian samples at Lima. This is consistent with the view that blood vessel proliferation is an important strategy in Ethiopian adaptation, insuring better blood flow to tissues and consequently better oxygen delivery.

Taken together these WBC gene-expression results give insight into the different ways in which Peruvians, Ethiopians and Himalayan highlanders adapt to life in chronic hypoxia.

## Discussion

The understanding of evolution depends on discerning the causes of variations among individuals which impact the fitness of future generations and its genetic basis.

The study of the biology of evolution is often confined to the laboratory and to model organisms. However, laboratory conditions, though offering advantages, are unlikely to accurately model the variations in environments that bear heavily on selection in wild populations. Thus, the study of fitness and the genetics that influence this are best carried out in matching environments to which humans are adapted. There is, therefore, great interest in transferring genomics to the field, away from the laboratory [12].

How humans adapted to ambient hypoxia through changes in the expression of hypoxia-related genes resembles a puzzle in which most pieces are missing. Here we provide a few of the missing pieces to strengthen the evidence that evolution over a relatively short time span has lead to survival advantages in Ethiopians, to a lesser extent in Himalayans, but not yet in Andeans who arrived at their present abode at a later date.

The study of human biology in the mountains began about 2000 years ago with a description by a Chinese official who wrote of a high-altitude traveler: “His face turns pale, his head aches and he begins to vomit” [13]. The Jesuit José de Acosta, a sixteenth century traveler in the Andes of Peru, was the first to attribute his discomfort, when reaching an altitude of 4802 meters, to the “thin air” [14].

Subsequently, numerous physiologic studies attempted to explain the effects of ambient hypoxia on humans, and prepare those who traveled to the mountains, not of necessity, but for pleasure and sport.

The strategy of field studies at altitude was inaugurated by T. H. Ravenhill who was the first to recognize in 1913, in a high altitude mining camp in Chile, the occurrence of acute mountain sickness, high altitude cerebral edema and high altitude pulmonary edema [15]. The first to describe chronic mountain sickness, as distinct from Ravenhill's acute mountain sickness, was Carlos Monge of Lima, Peru, who documented this affliction in 1928 in a native miner from Cerro de Pasco and continued to study chronic mountain sickness throughout his life [16]. The syndrome is characterized by widespread dysfunction attributed to severe hypoxia, especially during sleep, by accelerated aging and neurologic signs such as a decreased hypoxic ventilatory drive and a mild peripheral neuropathy [3], [17]

We have presented, for the first time, a comparison of hypoxia related gene expressions in WBC, in the three major high altitude populations, Andeans, Ethiopians and Himalayans, collected from the same individuals first in their native ambient hypoxia and then while normoxic. Previous population based studies, using physiologic measurements, have hinted at differences in human adaptive strategies encountered in the Andes, Himalayas and the East African high altitude plateau [6].

We first set out to uncover the molecular signature, defined here as a distinct pattern of gene expression, that underlies the frequent occurrence of CMS, a maladaptation syndrome in the Andes. We found that PDP2, a phosphatase that de-phophorylates the E1 alpha subunit of pyruvate dehydrogenase and promotes pyruvate entry into the Krebs cycle, is the best predictor of CMS. Low expression of mRNA encoding this enzyme may be a marker of chronic mountain sickness. We found, additionally, that in Cerro de Pasco, altitude 4338 m, where the molecular signatures of both study groups were influenced by the prevailing hypoxia, the CMS patients remained distinguishable from control highlanders (Fig 1).

The prevailing opinion that CMS is rare in Tibetans [18] was not borne out by our experience in Ladakh. We found, applying the CMS-scoring system developed for Andeans in Korzok, a village on the Tibetan plateau, situated at 4450 m., a surprisingly large number of subjects with CMS. The prevalence of the various symptoms and signs making up the CMS scores in the Himalayas was significantly different, however, from that found earlier in the Andes Fig. 4. This suggests that a better scoring system specifically developed for the Himalayas might uncover other populations living on the Tibetan plateau with comparable prevalence of CMS to that in the Andes.

The molecular signature of CMS in the Himalayas is surprisingly similar to that found in the Andes. Here too, PDP2 expression is the best predictor of CMS in ambient hypoxia (Fig. 2.). This underscores the robustness of our analyses in 2 continents and implies that CMS has a molecular signature that is, to some extent, dependent of genetic background. Both, Andeans and Himalayans are likely to have descended from the same ancestral population that migrated from Asia across ancient Beringea to the New World [19].

Homeostasis, the ability to maintain a steady state in the face of stress, is a fundamental property of life which presumably bestows evolutionary advantages to those who face life at altitude and its attendant, inescapable, hypoxic stress.

The low expression of mRNA encoding PDP2 in CMS patients but not in controls living at the same altitude in the Andes and Himalayas suggests that CMS represents a derangement in molecular homeostasis, with inadequate up-regulation of hypoxia-related genes to maintain a steady state in the face of hypoxic stress.

On the other hand, comparison of highland controls with Lima controls in Lima, where both groups were shown to be normoxic, revealed no differences in molecular signatures between these study groups (Fig 1). This supports the notion that our highland controls had no homeostatic derangement when hypoxia was no longer influencing their molecular signatures. These results were also consistent with clinical studies in Peru that showed that all symptoms and signs of CMS disappear after 2 months of sea level sojourn [20].

**Figure 6:**
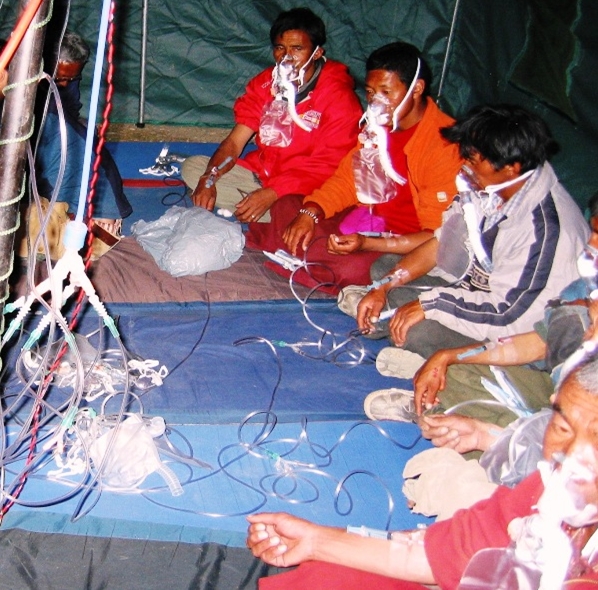
Hyperoxia in a tent at 4450 m. on the Tibetan plateau, near Korzok village in Ladakh, India. The subjects were exposed to hyperoxia for 1 hour. Note the venous catheters in place on the forearms to allow the drawing of blood for the post hyperoxia samples. This maneuver resulted in significant changes in gene expression in white blood cells.

Additionally, we examined molecular signatures in well-adapted East-African subjects and compared them to our highland and sea level subjects from Peru and Himalayan highlanders from the Tibetan plateau. In these field studies the limitations of geography precluded perfect matching of altitudes. Sea-level studies of the Andeans were performed in Lima at 150 m. whereas Ethiopians could only be brought down to an altitude of 794 m. in the Tekeze River Gorge. Also because of the difficulties of descent to the Gorge, the Ethiopians were studied after a much longer period of normoxia compared to the Peruvians. Moreover, because of similar constraints of geography, our subjects from Korzok village on the Tibetan plateau in Ladakh, were studied in artificial normoxia at their resident altitude of 4450 m. (Fig. 6)

Because of technical difficulties in East Africa and unavoidable delays only minute quantities of Ethiopian RNA were available, precluding the analysis of the entire set of genes examined in Peruvians and Himalayans.

The Ethiopian altitude dwellers had higher expression of PDP1 in their home village than Andean and Himalayan highlanders (Fig. 5). A change from hypoxia at altitude (88% saturation) to normoxia (96% saturation) in the Tekeze river Gorge did further increase the expression of this gene. All the other genes assayed at both locations remained unaffected by normoxia in Ethiopians and Himalayans (Fig 5). Thus, normal Tibetan plateau residents have a similar lack of responsiveness of gene expressions when made normoxic to that of Ethiopians (in a small subset of hypoxia related genes), supporting the impression that Himalayan highlanders occupy an intermediate niche in the “adaptive scale”, between Ethiopians (well adapted) and Andeans (least adapted).

Remarkably, the expression of most of the studied genes remained higher in Ethiopians in the Gorge than in our sea level Peruvian controls at Lima, except for PDK1. This is an enzyme thought to play a crucial role in tissue adaptation to hypoxia [21]. Its low expression in altitude adapted Ethiopians and Himalayans underscores their superior ability to cope with hypoxia, which may obviate the need to up-regulate PDK1 when in their native environment. (For glossary of terms see Text S1).

How might PDP2 expression levels protect against chronic mountain sickness? Recent studies on hypoxic cells have identified critical adaptations that could prevent hypoxia-induced increases in ROS production and thus forestall ROS damage to cellular proteins; also a damaging component of many hypoxia associated diseases at sea level such as Alzheimer's disease and other neurodegenerations.

Amongst the most notable adaptations is increased expression of PDK1 (PDH (pyruvate dehydrogenase) kinase 1) [21]. PDPs reverse the activity of PDKs, which limit glucose oxidation despite the presence of oxygen and maintain glycolysis. Thus the balance of activity between PDPs and PDKs determines whether organs oxidize glucose completely to CO2 and H2O, or whether they display “aerobic glycolysis”. Low PDP activity would favor aerobic glycolysis with reduced production of CO2. Thus the balance between hypoxic adaptation and CO2 mediated vasodilatation, to improve blood flow and thus oxygen availability to tissues, may be different in CMS patients.

The remarkable impact of PDP2 (tables 2, 3, 4) in predicting high CMS-scores in the Andes and Himalayas suggests that pyruvate metabolism which is critically influenced by PDP2 is disturbed in this disease and strengthens the proposition that low expression of some mitochondrial genes in response to hypoxia is important in the pathogenesis of CMS[21].

The niche graphs clearly illustrate the different molecular signatures of Andeans and Himalayans with and without CMS (Figs. 1, 2). The implication is that CMS is not simply a clinical manifestation of a homeostatic derangement but might signify that this derangement is superimposed on a genetic predisposition to this disease. CMS is known to be a disease of older men and post-menopausal women, in whom years of chronic hypoxia may have combined with an appropriate genetic background to precipitate the disorder.

Although no systematic survey to detect CMS has been conducted in Ethiopia, we and others have seen no evidence of this disorder on the East African high altitude plateau [22]. Our finding that at altitude, many genes examined here were expressed at levels that were several orders of magnitude higher in Ethiopians than in Peruvians supports earlier suggestions that Ethiopians show a different and more effective pattern of adaptation to life at altitude [6].

It is now recognized that one of the most important physiologic adjustment in the face of hypoxia is an increase in blood flow to tissues facilitated by vasodilatation [23]. One of the extensively studied mechanisms involves the release of endothelial nitric oxide synthase (eNOS) and the subsequent generation of NO. A failure to release NO, in sufficient amounts, has been blamed for a maladaptation syndrome in mountaineers [24]. NO diffuses from the endothelium to the vascular media activating guanylate cyclase which produces guanosine 3′ , 5′-monophosphate (cGMP). cGMP in turn activates protein kinase G (PKG) which is a key enzyme in vasorelaxation [23]. H_2_O_2_ and other ROS are also vasoactive and can modify other downstream vasorelaxants [10]. Thus, hypoxia and its vasodilator consequences are also dependent, to some extent, on ROS generation and on PKG1α.

In earlier studies, we examined the sensitivity of the cerebral circulation to hypoxia in the same Ethiopian subjects [25]. In these physiologic studies we found that the cerebral circulation of Ethiopians was insensitive to hypoxia, unlike in Peruvian high altitude dwellers, despite the responses of the cerebral circulation to exogenous NO donors being greater at altitude when compared to Peruvians, implying enhanced vasodilatation in response to hypoxia in Ethiopians [22].

However, Ethiopians had higher carbon dioxide levels (Table 1), and increased cerebral reactivity to carbon dioxide—both of which would likely promote increased cerebral blood flow, increasing oxygen delivery to the brain. The seemingly paradoxical insensitivity to hypoxia of the cerebral circulation in Ethiopian highlanders might therefore be advantageous to survival in the mountains if the pattern of carbon dioxide sensitivity of the cerebral circulation promotes better perfusion of the brain in Ethiopians when compared to Andeans [25].

Our Ethiopian subjects also lacked acute ventilatory responses to changes in PO2 at low and high altitude [25], [26].We interpreted this as indicating a survival advantage to altitude life involving anaerobic metabolism; advantageous in the highlands.

In the Himalayans the cerebral blood flow responses were similar to those we found in Ethiopians. They too have higher responses to CO2 than those reported for sea level subjects and their cerebral circulation is insensitive to hypoxia [27]. Moreover, we found also a blunted hypoxic ventilatory response and suggestions that other mechanisms in addition to hypoxia induced increases in ventilation, determine adaptation to “high-life” in the Himalayas [28].

In healthy sea-level individuals adaptive responses to hypoxia include increased production of vascular endothelial growth factors and other angiogenic cytokines [29]. Such responses are also found in Andeans and especially in those with CMS, where expression levels are very high when compared to sea level Peruvians [30]. We now find that well-adapted Ethiopians have even higher expression levels of these factors, which are known to promote tissue perfusion and better oxygen delivery. One key molecule, HIF-1 (hypoxia inducible factor-1), controls the expression of hundreds of genes, including the VEGFs and other angiogenic cytokines [29].

Here and in our previous studies we examined only white cells (WBC). Clinically, Andeans and Himalayans show evidence of vascular proliferation in the skin and conjunctivae [31]. These clinical signs were not present in our Ethiopian subjects. Thus it is conceivable that the angiogenic response to ambient hypoxia in Andeans and Himalayans affects primarily tissues such as the skin and conjunctivae, which does not boost oxygen delivery to vital tissues such as the brain; but it favors vital tissues, including the cardiovascular system, in the well-adapted East Africans; perhaps, a “tissue-specific” adaptation found only on the East African high altitude plateau.

HIF plays an important role in the induction of VEGF in response to hypoxia. However, induction of VEGF by hypoxia has recently been found to be also independent of HIF [32]. Instead, the transcriptional coactivator PGC-1α (peroxisome –proliferator-activated receptor-γ coactivator-1α) functions as a crucial regulator of mitochondrial function in response to hypoxic stress. Thus another adaptive strategy for human survival in chronic hypoxia could involve non-HIF dependent induction of VEGF and other angiogenic factors.

The insensitivity to hypoxia in Ethiopians and Himalayans, which we found in our previous physiologic studies, is now matched to an insensitivity in their molecular signatures to acute changes in blood oxygen tension.

Environmental challenges including hypoxia, which disturb homeostasis, can lead to disease onset or accelerated aging. These challenges are continuously met by changes in the biology of organisms. Proteostasis, the maintenance of cellular protein function, in the face of external and internal stresses is a key mechanism in the quest to counteract perturbations of the proteome (the proteins found in cells) [33].

There are numerous proteins that help organisms handle stress. Prominently, the heat–shock factors (HSF) which are also upregulated by hypoxic stress [34]. Some HSFs have found clinical utility in preventing hypoxic damage in neurodegenerations, cardiovascular, cerebrovascular and pulmonary diseases. And stimulating HSF has found application in experimental Parkinson's disease a disorder associated with ROS production [35]. By extension, increasing PDPs and PDK in chronic hypoxia might find application in hypoxia-associated diseases in clinics at sea level.

The confluence of results from molecular studies, physiologic investigations and clinical experience in Andeans, Ethiopians and Himalayans, suggests that further studies of these experiments of nature might soon find clinical utility at sea level.

## Methods

### Design

For an explanation of abbreviations see glossary of terms (Text S1).

A cross-sectional study was performed in Peru in three groups; high altitude natives with CMS; high altitude controls, and sea level people without high altitude ancestry. Lack of altitude ancestry was defined as absence of parents and grandparents native to the high Andes. Ten subjects were recruited in each highland group and 20 at sea level. The groups were matched by age and sex. The protocol was approved by the Ethics Committee of Cayetano Heredia University in Lima, Peru with Professor Dr. Luis Huicho MD, Universidad Nacional Mayor de San Marcos, Lima, Peru, as principal investigator.

A similar cross-sectional study was performed in Ethiopia on 8 high altitude native men born and resident in the Simen Mountain National Park, they were matched by age with the Peruvian subjects. The study was approved by the Ethiopian government and local research ethics committees with Dr. Guta Zenebe, Professor of Neurology, Addis Ababa University, School of Medicine and the Yehuleshet Higher Clinic, Addis Ababa, as principal investigator. (Approval RDHE/52-59/05 dated 31 January 2005, signed Yemane Teklai (Dr.), Head, Health Department and secretary National Ethiopian Research Council; NERC). All groups were studied at their native high altitudes and at low altitude in normoxia.

On the Tibetan plateau, in Ladakh, we obtained IRB approval from the Ladakh Institute of Prevention (For the Study of Environmental, Occupational, Life-Style related and High Altitude Diseases) Dr. Tensing Norboo, Honorary Secretary of the Institute, as principal investigator, dated April 15 2006. Because of the impossibility of taking Tibetans off the plateau to sea level in reasonable time we studied our subjects in their native environment and after 1 hour of hyperoxia at the same altitude.

A written informed consent was obtained from each subject in their native languages.

All studies were performed in accordance with the Declaration of Helsinki (2002) of the World Medical Association.

#### Inclusion criteria

Peruvian high altitude subjects were included providing they were native to and resident in Cerro de Pasco Peru, altitude 4338 m and did not drink herbal infusions that might reduce red cell mass; a frequent self administered remedy for CMS. We also included a matched group of subjects permanently resident at sea level in Lima without high altitude ancestry.

Ethiopian high altitude subjects were included providing they were permanent residents and natives of Chennek village situated at 3662 m altitude in the Simen Mountain National Park, Ethiopia.

Himalayan subjects were included if they were natives of Korzok village altitude 4450 m. in Ladakh, India.

#### Exclusion criteria

We excluded subjects with significant acute or chronic clinical conditions, or those that had visited altitudes below 2000 m in the 3 months preceding the study. Peruvian subjects were examined in Cerro de Pasco and in Lima by a senior Peruvian physician (LH). All Ethiopians were examined by 3 Ethiopian physicians, who accompanied us from Addis Ababa, to exclude disorders that might confound gene expression.

Himalayan subjects were examined by 2 physicians from Leh, Ladakh (TN and DD) who accompanied us to Korzok and by one of us (OA) with the assistance of the local physicians.

### Study procedures

High altitude Peruvian subjects were studied in Cerro de Pasco first, and then they were transferred to Lima (150 m.) in approximately 6 hours by bus. Sea level natives were studied in Lima.

Ethiopians were first studied near their permanent high altitude residence. They were then transferred to low altitude. It took approximately 12 hours to arrive by truck in the Tekeze River Gorge (794.). A clinical history and results of physical examination and arterial oxygen saturation (pulse oximetry (SaO_2_) were recorded. The medical history, in Peru, included current and past diseases, use of cytotoxic drugs or herbal infusions for reducing polycythemia, “maca” (a plant protein supplement frequently used in Peru, containing numerous plant sterols), and history of descent to altitudes below 2000 m.

A chronic mountain sickness score (CMS-sc) was obtained for each high altitude subject at the high altitude locations in Peru, Ethiopia and in Korzok village using an internationally accepted scoring system. A score of ≥12 is considered to indicate CMS [11].

Venous blood samples were taken for hemoglobin and hematocrit determination, and for RNA assays. Hemoglobin was determined by spectrophotometry and hematrocrit by a standard capillary technique. At sea level, blood was taken from high altitude subjects within 1 hour after arrival at Lima. In Ethiopian subjects, venous blood samples were obtained the morning after arrival (∼12 hours of normoxia).

The Himalayans were studied first in ambient air at 4450 m. and then after exposure for 1 hour to hyperoxia at the same altitude (Fig. 6). Blood samples were obtained before and after 1 hour of hyperoxia.

We made our subjects hyperoxic entering them into the protocol at 5–10 min intervals. As each subject was seated in our research tent, a 22 G indwelling intravenous catheter was inserted into a vein in the dorsum of the hand or forearm and taped in place. Blood was drawn and immediately transferred to appropriate tubes for later RNA analysis. The tape securing the catheter was labeled with subject identification number, test tube identification, and time of blood sampling. The intravenous catheter was then flushed with normal saline and capped. Subjects immediately took their place in a semicircle surrounding the single K-size O_2_ tank and had a highly efficient [36] sequential gas delivery oxygen mask (Hi-Ox^80^ ViasysHealthCare, Yorba Linda CA, USA) placed on their face [37] (Fig. 6). Oxygen flow was adjusted such that end-tidal PO_2_ was between 100 and 110 mmHg. The O_2_ mask of each succeeding subject was connected in a branching fashion to the O_2_ tank by using a 3-way tubing connector. With each succeeding branching of the tubing, the gas flow from the tank was increased and the end-tidal PO_2_ for each subject was re-checked. This was continued until a maximum of 8 subjects were being administered O_2_. After one hour a second set of blood samples was drawn and new subjects were entered into the hyperoxia part of the protocol. This experimental set-up required only 3 investigators, one to insert venous cannulae and draw control blood samples; one to adjust the gas flows to the Hi-Ox^80^ and confirm end tidal values; and one to draw the blood samples at the conclusion of the hyperoxic period. The average O_2_ flow per subject was about 0.5 L/min. About one third of the O_2_ tank was consumed during the experiment.

### Quantitative real-time PCR

The total RNA of human white blood cells was extracted using the PAXgene Blood RNA kit (Qiagen, Germany) following the manufacturer's protocol. One microgram (1 μg) of total RNA was reverse transcribed into first-strand cDNA using the RETROscript reverse transcriptase kit and oligo dT primers (Ambion, TX) according to the manufacturer's protocols. One microliter (1 μl) of cDNA from the reverse transcriptase reaction was used as the template for quantitative real-time PCR reaction with a final PCR reaction volume of 25 μl, with the 5′ and 3′ gene specific PCR primer concentrations at 100 nM each. PCR primers were designed using Primer3 software (Whitehead Institute, MIT, MA) according to the coding sequences of each gene. Quantification of mRNA expression was performed (in triplicate) using the SYBR Green SuperMix (BioRad, CA) and a 2-step PCR reaction procedure, performed on the MyiQ Single Color Real-Time PCR Detection System (BioRad, CA). In brief, after the initial denaturation at 95° C for 3 min, 45 cycles of primer annealing and elongation were conducted at 58° C for 45 seconds, followed by denaturation at 95° C for 10 s. Fluorescent emission data were captured, and mRNA levels were quantified using the threshold cycle value. To compensate for variations in input RNA amounts and efficiency of reverse transcription, data for each target gene mRNA of each sample were normalized by reference to the data obtained for the house keeping HPRT (GenBank accession no. X62085) determined from the same sample. Each real-time PCR assay was repeated twice.

### Statistical methods

Gene expression data were log transformed to obtain symmetric distributions for analysis and for graphical purposes. Comparison of gene expression values between the two altitudes were by paired t-tests. The gene expression variables that best predicted the CMS classification of 10 Cerro de Pasco subjects with CMS (CMS score> = 12) and 10 Cerro de Pasco controls were obtained by considering 1 and 2 variable models in stepwise and “all subsets” logistic regression, sensitivity and specificity using median cut scores, and the effect sizes (standardized beta) in univariate and bivariate logistic regressions at sea level (Lima) and at altitude (Cerro de Pasco, 4338m.). The best two gene model using expressions at sea level (Lima) was LDHA based on sensitivity (90%) and specificity (100%) and PDP2 based on effect size (standardized beta) in logistic regression. Since LDHA was not measured at high altitude (Cerro), PDK3 was used based on having the highest correlation with LDHA at Lima. The “niche” scatter plots show the two predictor genes selected as best at each altitude (Fig. 1).

The impact table is constructed using CMS scores as a continuous variable and consists of the effect sizes (standardized beta) for each gene in univariate linear regression in the first columns, the effect sizes for each gene adjusting for the best univariate predictor (PDP2 at both altitudes) in the middle columns, and the residual effect size of PDP2 adjusting for each of the other genes in the final columns (Table 2,3). In addition to the comparison among high altitude groups, the Lima control subjects are compared to the high altitude control group, while in Lima (Fig. 1).

The data from the Himalayans were analyzed in similar fashion (Fig. 2, Table 4, 5).

The log transformed gene expressions for the Ethiopian subjects (n = 8) for the 8 genes measured in common with the 2 other high altitude populations and Lima control subjects are compared as line graphs (signatures) as a function of the gene number (in arbitrary order) (Fig. 5). Comparison between altitudes is by paired t-tests and between groups by unpaired t-tests.

### Methodological Constraints

We studied our subjects in the field in 3 continents over a period of 3 years. Strict laboratory procedures were, usually, unattainable. Although larger numbers of subjects in each study group were desirable these could not be assembled because of time limitations at altitude for sea level researchers and especially because molecular studies were combined with physiologic and clinical evaluations of the same individuals.

The research station of the Universidad Peruana Cayetano Heredia in Cerro de Pasco, Peru maintains a list of CMS patients and controls. We used this list to recruit our subjects in Peru. Because women are protected from CMS until the menopause the list contains mainly men. Thus, the results of our initial Peruvian study had unavoidable gender bias. Subsequently, the gender bias imposed in Peru constrained our selection of subjects in Ethiopia and the Himalayas.

The geography in the 3 continents precluded exact matching of altitudes in each location. The choice of our research sites was also influenced by political expediency, safety issues and ease of access.

In previous field studies we found that exposure to 1 hour of normoxia at sea level was sufficient to change gene expression in white cells. This, partly, inspired the design of our study in the Himalayas where 1 hour of hyperoxia proved sufficient to change some, but not all, gene expressions without a change in location. We were encouraged that our method was effective because this manipulation (Fig. 6) led to significant changes in gene expression levels in 7 of 20 genes assayed in Himalayan CMS patients (PDK3, PDK4, PDP1, PDP2, PDHE1A1, HIF1b, and HPH2), in 3 genes (PDK3, PDK4, and PDP1) when all subjects were considered as a group, but no changes in expression levels in Himalayan control subjects.

Our analyses of Ethiopian gene expression were limited by the vagaries of transport, facilities and access to laboratory personnel, typical of field studies. Thus, only minute quantities of a small subset of genes were eventually available for comparison between the 3 high altitude populations.

In Ethiopia and Peru, the travel time necessary to achieve normoxia varied greatly. Nevertheless, significant changes in expression of hypoxia related genes were found in Peru. The Ethiopians, though spending the longest time in normoxia, failed, mostly, to show measurable changes in their gene expression induced by descent.

The number of subjects was small; we emphasize this study is preliminary rather than conclusive. But the results are suggestive and consistent enough with physiologic and epidemiologic studies to warrant further investigation of these altitude populations.

## Supporting Information

Text S1 Glossary of terms
(0.03 MB DOC)

## References

[1] Pennisi E (2008). Are epigenticists ready for big science?. Science.

[2] Cohen J (2007). Genomics. DNA duplications and deletions help determine health.. Science.

[3] Thomas PK, King RH, Feng SF, Muddle JR, Workman JM (2000). Neurological manifestations in chronic mountain sickness: the burning feet-burning hands syndrome.. J Neurol Neurosurg Psychiatry.

[4] Niermeyer S, Zamudio S, Moore LG, Hornbein TF, Schoene RB (2001). The People.. High altitude: An exploration of human adaptation.

[5] Beall CM (2007). Two routes to functional adaptation: Tibetan and Andean high-altitude natives..

[6] Beall CM, Decker MJ, Brittenham GM, Kushner I, Gebremedhin A (2002). An Ethiopian pattern of human adaptation to high-altitude hypoxia.. Proc Natl Acad Sci U S A.

[7] Beall CM (2007). Detecting natural selection in high-altitude human populations.. Respiratory physiology & neurobiology.

[8] Bakonyi T, Radak Z (2004). High altitude and free radicals.. Journal of Sports Science and Medicine.

[9] Hartzell HC (2007). Cell biology. The stress of relaxation.. Science.

[10] Burgoyne JR, Madhani M, Cuello F, Charles RL, Brennan JP (2007). Cysteine redox sensor in PKGIa enables oxidant-induced activation.. Science.

[11] Leon-Velarde F, McCullough RG, McCullough RE, Reeves JT (2003). Proposal for scoring severity in chronic mountain sickness (CMS). Background and conclusions of the CMS Working Group.. Advances in experimental medicine and biology.

[12] Ellegren H, Sheldon BC (2008). Genetic basis of fitness differences in natural populations.. Nature.

[13] Gilbert DL (1983). The first documented report of mountain sickness: the China or Headache Mountain story.. Respiration physiology.

[14] Gilbert DL (1983). The first documented description of mountain sickness: the Andean or Periacaca story.. Respiration physiology.

[15] Sutton JR (1983). Classification and terminology of altitude illnesses.. Semin Respir Med.

[16] Monge-M C (1928). La Enfermedad de los Andes.. Anales de la Facultad de Medicina de Lima.

[17] Sime F, Monge C, Whittembury J (1975). Age as a cause of chronic mountain sickness (Monge's disease).. International journal of biometeorology.

[18] Monge CC, León-Velarde FS (2003). El Reto fisiólogico de vivir en los Andes.. Travaux de l'Institut Française d'Études Andines vol. 177 pp. 435 chapter 13.

[19] Goebel T, Waters MR, O'Rourke DH (2008). The late Pleistocene dispersal of modern humans in the Americas Science..

[20] Hurtado A (1942). Chronic Mountain Sickness.. J Am Med Assoc.

[21] Semenza GL (2007). Oxygen-dependent regulation of mitochondrial respiration by hypoxia-inducible factor 1.. The Biochemical journal.

[22] Appenzeller O, Claydon VE, Gulli G, Qualls C, Slessarev M (2006). Cerebral vasodilatation to exogenous NO is a measure of fitness for life at altitude.. Stroke; a journal of cerebral circulation.

[23] Singel DJ, Stamler JS (2004). Blood traffic control.. Nature.

[24] Busch T, Bartsch P, Pappert D, Grunig E, Hildebrandt W (2001). Hypoxia decreases exhaled nitric oxide in mountaineers susceptible to high-altitude pulmonary edema.. Am J Respir Crit Care Med.

[25] Claydon VE, Gulli G, Slessarev M, Appenzeller O, Zenebe (2008). Cerebrovascular Responses to Hypoxia and Hypocapnia in Ethiopian High Altitude Dwellers.. Stroke; a journal of cerebral circulation.

[26] Slessarev M, Claydon VE, Gulli G, Dufour S, Appenzeller O, Roach RC, Wagner PD, Hackett PH (2007). Altered metabolic state defines adaptation to high altitude in Ethiopian highlanders.. Advances in experimental medicine and biology..

[27] Slessarev M Prisman E, Ito S, Watson R, Preiss D, Roach RC, Wagner PD, Hackett PH (2007). The cerebral blood flow response to CO2 in Himalayan highlanders with and without chronic mountain sickness.. Hypoxia and the circulation. Advances in experimental medicine and biology..

[28] Slessarev M, Prisman E, Ito S, Watson R, Jensen D, Roach RC, Wagner PD, Hackett PH (2007). Chemoreflex control of breathing in Himalayan and sea level residents.. Hypoxia and the circulation. Advances in experimental medicine and biology..

[29] Semenza GL (2007). Life with oxygen.. Science.

[30] Appenzeller O, Minko T, Pozharov V, Bonfichi M, Malcovati L (2003). Gene expression in the Andes; relevance to neurology at sea level.. Journal of the neurological sciences.

[31] Appenzeller O, Minko T, Qualls C, Pozharov V, Gamboa J (2006). Gene expression, autonomic function and chronic hypoxia:lessons from the Andes.. Clin Auton Res.

[32] Arany Z, Foo S-Y, Ma Y, Ruas JL, Bommi-Reddy A (2008). HIF-indepenedent regulation of VEGF and angiogenesis by the transcriptional coactivtor PGC-1α.. Nature.

[33] Balch WE, Morimoto RI, Dillin A, Kelly JW (2008). Adapting protoestasis for disease intervention.. Science.

[34] Sartori C, Scherrer U, Roach RC, Wagner PD, Hackett PH (2003). Turning up the heat in the lungs. A key mechanism to preserve their function.. Advances in experimental medicine and biology..

[35] Shen HY, He JC, Wang Y, Huang QY, Chen JF (2005). Geldanamycin induces heat shock protein 70 and protects against MPTP-induced dopaminergic neurotoxicity in mice.. The Journal of biological chemistry.

[36] Slessarev M, Fisher JA (2006). Oxygen Administration in the ED: Choosing the appropriate dosage and technology.. Israeli Journal of Emergency Medicine.

[37] Slessarev M, Somogyi R, Preiss D, Vesely A, Sasano H (2006). Efficiency of oxygen administration: sequential gas delivery versus “flow into a cone” methods.. Crit Care Med.

