# Correction: Adaptation and Mal-Adaptation to Ambient Hypoxia; Andean, Ethiopian and Himalayan Patterns

**DOI:** 10.1371/annotation/aba20dc1-b10d-464c-9671-c47b956d1718

**Published:** 2008-06-06

**Authors:** Guoqiang Xing, Clifford Qualls, Luis Huicho, Maria Rivera-Ch, Tsering Stobdan, Marat Slessarev, Eitan Prisman, Shoji Ito, Hong Wu, Angchuk Norboo, Diskit Dolma, Moses Kunzang, Tsering Norboo, Jorge L. Gamboa, Victoria E. Claydon, Joseph Fisher, Guta Zenebe, Amha Gebremedhin, Roger Hainsworth, Ajay Verma, Otto Appenzeller

The eighth author's name appears incorrectly. It should be: Shoji Ito

