# Supplementary material for: Adaptation and Mal-Adaptation to Ambient Hypoxia; Andean, Ethiopian and Himalayan Patterns
Source: PLoS One. 2008 Jun 4;3(6):e2342. doi: 10.1371/journal.pone.0002342 (PMC2396283; doi:10.1371/journal.pone.0002342)
Supplement: Text S1 — Glossary of terms (0.03 MB DOC) [file pone.0002342.s001.doc]

# On line supplementary information for:

**ADAPTATION AND MAL**-**ADAPTATION TO AMBIENT HYPOXIA; ANDEAN, ETHIOPIAN AND HIMALAYAN PATTERNS.**

**GUOQIANG XING et al.**

**GLOSSARY OF TERMS.**

**CMS**=Chronic Mountain Sickness. A neurologic disorder appearing in men in their thirties and in post-menopausal women, characterized by neurologic symptoms and signs, extreme cyanosis, injected conjunctivae, dilated veins, pulmonary hypertension, right sided heart failure, severe, mostly nocturnal, hypoxia and excessive polycythemia. Death is usually from right heart failure. All symptoms and signs disappear after about 2 months at sea level.

**CMS-score=** Points, from 1-3, are assigned to symptoms and signs. The aggregate of accumulated points forms the CMS-score. ≤12=normal; 12-18=mild; 19-24=moderate; >25=severe CMS. (from manuscript reference # 11)

**Molecular signature**= A distinct pattern of gene expression.

***EPO***= Erythropoietin gene. Transcriptional control is mediated by HIF. Involved in erythropoiesis.

**HPH1,HPH2, HPH3**= HIF prolyl hydroxylases (HPH). Enzymes that allow oxygen tension to control HIF-alpha protein levels.

**VEGFC**= Vascular endothelial growth factor, the C-form was measured.

**PDK1, PDK2, PDK3, PDK4**=. Pyruvate dehydrogenase Kinase. PDK2 is the most widely expressed form of PDK. These kinases are involved in "aerobic glycolysis" (the Warburg effect). They support a metabolic pattern seen in many (but not all) hypoxia adapted tissue, also in cancer cells and activated immune cells.

**HIF1A, HIF2A, HIF3A, HIF1B**= Composed of alpha and beta subunits. Only the alpha subunit protein levels are regulated by oxygen. HIF1B is used as a dimerizing partner by all three HIF1A’s.

**PDP2,1**= Phosphatases that de-phosphorylates the E1 alpha subunit of pyruvate dehydrogenase. The enzymes encoded by this gene attempt to reverse the de- phosphorylation activity of PDKs and promote pyruvate entry into the Krebs cycle.

**PDHE1A1**= the gene encodes the E1 alpha subunit of pyruvate dehydrogenase (PDH), which is the regulatory component of PDH and the target of PDKs and PDPs.

**GAPDH**= Glyceraldehyde phosphate dehydrogenase. Regulated by HIF. GAPDH has a central position in the glycolytic cascade.

**EPOR**= Erythropoietin receptor.

**GLUT1**= Glucose transporter protein 1. GLUT1 is known to be one glucose transporter sensitive to hypoxia and HIF1; it is widely expressed in most tissues.

**LDHA**= Lactate dehydrogenase A. LDHA is involved in "buffering" pyruvate by reversibly converting to lactate. It favors formation of lactate, when pyruvate builds up too quickly or when there is a block in pyruvate metabolism due to pyruvate dehydrogenase inhibition or lack of sufficient oxygen.

**CATD**= Cathepsin-D. CATD is a protease involved in extracellular matrix modification and tissue invasion. It is a HIF1 regulated gene relevant to the biology of many invasive cells but has other functions as well.
